# Endoscopic Combined Intrarenal Surgery: best practices and future perspectives

**DOI:** 10.1590/S1677-5538.IBJU.2024.9921

**Published:** 2024-08-25

**Authors:** Anderson B. Pellanda, Fabio C. M. Torricelli, John Denstedt, Alexandre Danilovic, Giovanni S. Marchini, Fabio C. Vicentini, Carlos A. Batagello, William C. Nahas, Eduardo Mazzucchi

**Affiliations:** 1 USP Faculdade de Medicina da Universidade de São Paulo Departamento de Cirurgia São Paulo SP Brasil Divisão de Urologia, Departamento de Cirurgia, Faculdade de Medicina da Universidade de São Paulo - USP, São Paulo, SP, Brasil; 2 University of Western Ontario in London Canada University of Western Ontario in London, Canada

**Keywords:** Kidney, Lithotripsy, Urinary Calculi

## Abstract

**Introduction:**

Endoscopic Combined Intrarenal Surgery (ECIRS) has emerged as a promising technique for the management of large and complex kidney stones, potentially offering advantages over traditional Percutaneous Nephrolithotomy (PCNL). This study aims to evaluate best practices, outcomes, and future perspectives associated with ECIRS.

**Materials and Methods:**

A comprehensive PubMed search was conducted from 2008 to 2024, using MESH terms and the following key words: "ECIRS" and "Endoscopic Combined Intrarenal Surgery" The search yielded 157 articles, including retrospective cohort studies, two randomized controlled trials (RCTs), and four meta-analyses comparing ECIRS with PCNL. Most important findings were summarized regarding indications, patient positioning, kidney access, tract size, surgical outcomes, and complications.

**Results:**

ECIRS demonstrated higher stone-free rate, lower complication rate, and a reduced need for multiple procedures compared to traditional PCNL. Additionally, ECIRS has the potential to integrate new technologies to further enhance outcomes.

**Conclusion:**

ECIRS demonstrates significant advantages in the management of large kidney stones. Future research should focus on well-designed RCTs to provide robust evidence of its efficacy, safety, and cost-effectiveness, potentially establishing ECIRS as the first option treatment for complex kidney stones.

## INTRODUCTION

Complex and large kidney stones pose a significant challenge in urology, necessitating a careful balance between effectiveness and safety when selecting the optimal surgical approach. Prior to the development of endoscopic and percutaneous techniques, open and laparoscopic surgeries were commonly utilized, yielding good outcomes in stone clearance but also carrying high morbidity. Since its initial description by Fernstrom in 1976 ( 1 ), percutaneous nephrolithotomy (PCNL) has emerged as the gold standard treatment modality for large kidney stones (>2cm) ( 2 , 3 ). Over the past decades, PCNL has undergone numerous advancements and refinements. These include enhancements in patient positioning ( 4 – 6 ), improvements in kidney puncture guidance ( 7 – 9 ), advancements in energy delivery systems ( 10 , 11 ), development of effective suction devices ( 11 , 12 ), and utilization of flexible ( 13 – 15 ) and miniaturized instruments ( 16 ).

Among these innovations, the integration of retrograde flexible nephroscopy with standard PCNL stands out significantly. This approach facilitates surgeon access to all calices ( 14 ) and reduces the requirement for aggressive kidney instrumentation ( 13 ), leading to improved outcomes ( 17 ). Despite recommendations for routine use of flexible scopes alongside standard PCNL ( 2 ), many studies still report the exclusive use of rigid nephroscopes ( 18 , 19 ). Flexible ureteroscopes have supported percutaneous procedures since 1995 ( 20 ). However, it was not until 2008 that Scoffone et al. ( 21 ) introduced the term Endoscopic Combined Intrarenal Surgery (ECIRS) to describe the simultaneous use of rigid nephroscopy and retrograde flexible ureteroscopy. Subsequently, several studies have aimed to compare traditional PCNL with ECIRS, but high-quality research is needed to establish ECIRS as the new standard treatment for large kidney stones ( 3 , 18 , 22 – 25 ).

ECIRS presents distinct features and challenges. One notable concern is the requirement for two surgeons and two video systems, which can pose logistical and financial burdens, particularly in settings with limited resources. Moreover, the cost-effectiveness of this simultaneous endoscopic approach remains uncertain, prompting questions about its economic viability. The complexity of ECIRS, which involves both antegrade and retrograde accesses, demands considerable skill and coordination, thereby limiting its broader adoption.

Despite these challenges, ECIRS offers potential benefits that makes it an attractive option for treating large kidney stones. These include a high stone-free rate, lower morbidity, and fewer procedures required per patient to achieve the surgical goal. The ability of ECIRS to access all calices using flexible instruments and its potential to minimize kidney trauma can lead to improved patient outcomes. This includes reduced complication rate and faster recovery time compared to traditional approaches.

This study aims to discuss the best practices in surgical techniques and present the outcomes associated with ECIRS in the management of large kidney stones. By critically analyzing the available evidence, our goal is to assess whether the advantages of ECIRS outweigh its drawbacks. This will provide valuable insights for urologists considering ECIRS as a treatment option for their patients.

## DATA ACQUISITION

**Figure 1:**
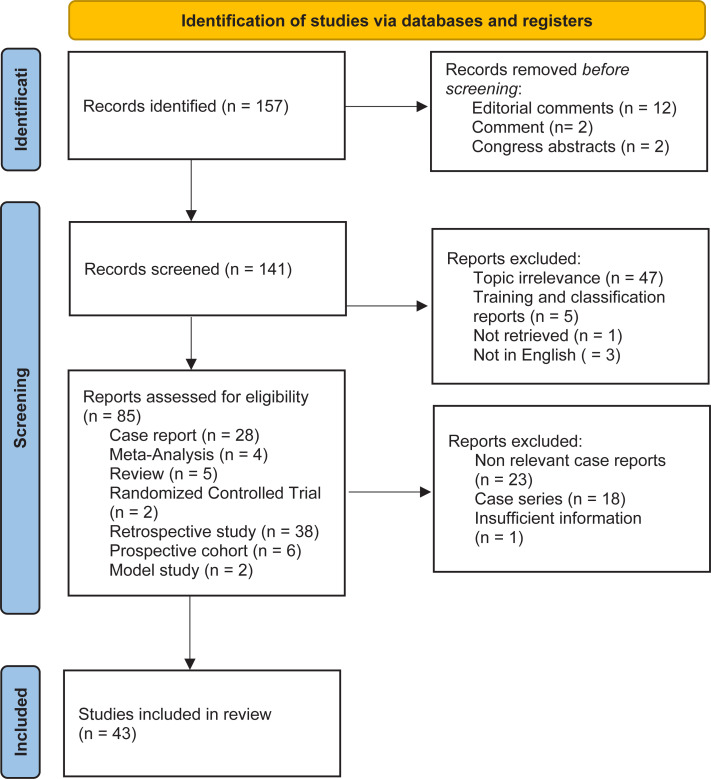
Flowchart.

We conducted an extensive PubMed search covering the period from 2008 to 2024, using MESH terms and key words such as "ECIRS" and "Endoscopic Combined Intrarenal Surgery" ( Figure-1 ).

Our PubMed search yielded 157 articles on ECIRS. Among these, most were retrospective cohort studies. There were only two prospective randomized controlled trials (RCTs) identified: one RCT compared the efficacy and safety of mini-ECIRS versus a combination of PCNL and mini-PCNL for treating staghorn calculi ( 18 ); the second RCT examined the outcomes of mini-ECIRS in different patient positions ( 26 ). Despite the limited number of RCTs, four meta-analyses were published in 2022 ( 22 – 25 ), comparing ECIRS with PCNL. These systematic reviews encompassed a variety of study designs, including retrospective studies with both supine and prone patient positioning, and evaluations of both standard and miniaturized ECIRS techniques. The summaries of these meta-analyses provided insights into several critical aspects of ECIRS versus PCNL, including efficacy, safety, and procedural outcomes. These findings are essential for understanding the comparative effectiveness of ECIRS in managing large kidney stones and can guide clinical decision-making in urology.

Table 1 and 2 summarize data from meta-analyses.

**Table 1:** Data from Meta-Analyses

Patient positioning									
Meta Analysis	Patients (n)	Studies included	Type of study	ECIRS	PCNL	ECIRS (n)	PCNL (n)	Tract size	Objective comparison
Abdullatif et al. 2022 ( 25 )	546	Wen, et al. 2016 ( 18 )	RCT	GMSV	Prone	33	34	20 Fr	mini-ECIRS vs mini-PCNL
		Nuño de la Rosa, et al. 2014 ( 50 )	Retrospective	GMSV	Supine	73	98	24-30 Fr	ECIRS vs PCNL
		Hamamoto, et al 2014 ( 19 )	Retrospective	Prone splitleg	Prone	60	101	18 Fr (mini) / 30 Fr (PCNL)	mini-ECIRS vs mini-PCNL vs PCNL
		Leng, et al. 2018 ( 51 )	Retrospective	Oblique supine lithotomic	Oblique supine lithotomic	44	43	16-18 Fr	mini-ECIRS vs mini-PCNL
		Zhao, et al. 2021 ( 52 )	Retrospective	GMSV	Prone	66	74	16-18 Fr	mini-ECIRS vs mini-PCNL
Widyokirono et al. 2022 ( 22 )	614	Wen, et al. 2016 ( 18 )	RCT	GMSV	Prone	33	34	20 Fr	mini-ECIRS vs mini-PCNL
		Nuño de la Rosa, et al. 2014 ( 50 )	Retrospective	GMSV	Supine	73	98	24-30 Fr	ECIRS vs PCNL
		Hamamoto, et al. 2014 ( 19 )	Retrospective	Prone splitleg	Prone	60	101	18 Fr (mini) / 30 Fr (PCNL)	mini-ECIRS vs mini-PCNL vs PCNL
		Leng, et al. 2018 ( 51 )	Retrospective	Oblique supine lithotomic	Oblique supine lithotomic	44	43	16-18 Fr	mini-ECIRS vs mini-PCNL
		Zhao, et al. 2021 ( 52 )	Retrospective	GMSV	Prone	66	74	16-18 Fr	mini-ECIRS vs mini-PCNL
		Kontos, et al. 2018 ( 53 )	Retrospective	Supine	Supine	33	35	NA	ECIRS vs PCNL
Liu et al. 2022 ( 23 )	919	Wen, et al. 2016 ( 18 )	RCT	GMSV	Prone	33	34	20 Fr	mini-ECIRS vs mini-PCNL
		Nuño de la Rosa, et al. 2014 ( 50 )	Retrospective	GMSV	Supine	73	98	24-30 Fr	ECIRS vs PCNL
		Hamamoto, et al. 2014 ( 19 )	Retrospective	Prone splitleg	Prone	60	101	18 Fr (mini) / 30 Fr (PCNL)	mini-ECIRS vs mini-PCNL vs PCNL
		Leng, et al. 2018 ( 51 )	Retrospective	Oblique supine lithotomic	Oblique supine lithotomic	44	43	16-18 Fr	mini-ECIRS vs mini-PCNL
		Zhao, et al. 2021 ( 52 )	Retrospective	GMSV	Prone	66	74	16-18 Fr	mini-ECIRS vs mini-PCNL
		Isac, et al. 2013 ( 54 )	Retrospective	Prone splitleg	Prone	62	96	30 Fr	Endoscopic-guided versus fluoroscopic-guided renal access in PCNL
		Xu, et al. 2019 ( 55 )	Retrospective Meeting abstract	NA	NA	61	74	16-22 Fr	mini-ECIRS vs mini-PCNL
Gauhar et al. 2022 ( 24 )	2054	Wen, et al. 2016 ( 18 )	RCT	GMSV	Prone	33	34	20 Fr	mini-ECIRS vs mini-PCNL
		Nuño de la Rosa, et al. 2014 ( 50 )	Retrospective	GMSV	Supine	73	98	24-30 Fr	ECIRS vs PCNL
		Hamamoto et al. 2014 ( 19 )	Retrospective	Prone splitleg	Prone	60	101	18 Fr (mini) / 30 Fr (PCNL)	mini-ECIRS vs mini-PCNL vs PCNL
		Leng, et al. 2018 ( 51 )	Retrospective	Oblique supine lithotomic	Oblique supine lithotomic	44	43	16-18 Fr	mini-ECIRS vs mini-PCNL
		Zhao, et al. 2021 ( 52 )	Retrospective	GMSV	Prone	66	74	16-18 Fr	mini-ECIRS vs mini-PCNL
		Isac, et al. 2014 ( 54 )	Retrospective	Prone splitleg	Prone	62	96	30 Fr	Endoscopic-guided versus fluoroscopic-guided renal access in PCNL
		Mami, et al. 2021 ( 56 )	Retrospective	Prone	Prone	18	52	NA	ECIRS vs PCNL vs RIRS
		Kawahara, et al. 2012 ( 57 )	Retrospective	GMSV	Prone	27	23	24-30 Fr	Endoscopic-guided versus ultrasound- guided renal access in PCNL
		Hong, et al. 2016 ( 58 )	Retrospective	GMSV	Prone	78	90	> 20 Fr	ECIRS vs PCNL
		Gao, et al. 2019 ( 59 )	Retrospective	Prone splitleg	Prone	45	40	18 Fr	mini-ECIRS vs RIRS vs miniPCNL
		Xu, et al. 2019 ( 55 )	Retrospective Meeting abstract	NA	NA	61	74	16-22 Fr	mini-ECIRS vs mini- PCNL
		Beck, et al. 2009 ( 60 )	Retrospective Meeting abstract	NA	NA	51	70	NA	Endoscopic-guided renal access in PCNL
		Zelvys, et al. 2014 ( 61 )	Retrospective Meeting abstract	Supine	Supine or prone	22	113	NA	ECIRS vs PCNL
		Zhang, et al. 2016 ( 62 )	Retrospective Meeting abstract	NA	NA	84	197	NA	Supermini-ECIRS vs mini-PCNL
		Yong, et al. 2017 ( 63 )	Retrospective Meeting abstract	Supine	Supine or prone	16	91	NA	ECIRS vs PCNL
		Kavaliauskaite, et al. 2018 ( 64 )	Retrospective Meeting abstract	NA	NA	37	93	NA	ECIRS vs PCNL

ECIRS = Endoscopic Combined Intrarenal Surgery; PCNL = percutaneous nephrolithotomy; mini-PCNL = miniaturized percutaneous nephrolithotomy; mini-ECIRS = miniaturized Endoscopic Combined Intrarenal Surgery; RCT = Randomized Controled Trial; GMSV = Galdakao-modified supine Valdivia; Fr = French

**Table 2:** Outcomes from Meta-Analyses

Result									
Meta-Analysis	SFR	Operative time	Blood loss	Transfusions	Complications	Hospital Stay	Sepsis	Fever	Auxiliary procedures
Abdullatif et al. 2022 ( 25 )	Favors ECIRS	NS	NS	NS	Favors ECIRS	Favors ECIRS	NA	NA	NA
Widyokirono et al. 2022 ( 22 )	Favors ECIRS	NS	NS	NA	Favors ECIRS	NA	Favors ECIRS over PCNL / = mini- PCNL	NA	Favors ECIRS
Liu et al. 2022 ( 23 )	Favors ECIRS	NS	NS	Favors ECIRS	Favors ECIRS	NS	NA	NS	NA
Gauhar et al. 2022 ( 24 )	Favors ECIRS *	NS	Favors ECIRS	NS	NA	NS	NS	NS	Favors ECIRS

* Forrest plot table favors ECIRS, but plot diagram is inverted;

NS = not statistically significant; NA = data not available; ECIRS = Endoscopic Combined Intrarenal Surgery; PCNL = percutaneous nephrolithotomy; mini-PCNL = miniaturized percutaneous nephrolithotomy

## INDICATIONS

ECIRS shares similar indications with PCNL but offers the potential benefit of reducing the number of percutaneous tracts required to manage large or complex kidney stones ( 27 ). Moreover, ECIRS may present advantages in specific clinical scenarios, including:

1. Pediatric patients ( 28 )
2. Transplanted kidney ( 29 )
3. Management of encrusted ureteral stents ( 30 )
4. Treatment of large ureteral stones ( 31 )
5. Simultaneous management of renal and ureteral stones ( 32 )
6. Treatment of upper urinary tract urothelial carcinoma ( 33 )

These specialized applications highlight the versatility of ECIRS across various challenging urological conditions, underscoring its potential as a preferred or complementary approach in specific patient populations and clinical settings.

## POSITIONING AND PREPARATION OF THE PATIENT

Initially, ECIRS was described in the Galdakao-modified supine Valdivia (GMSV) position ( 4 , 21 ). Over time, various alternative patient positions have been explored, including:

- **Prone Split-Leg Position:** This position involves placing the patient prone with the legs split apart, facilitating access to the kidney and improving stone clearance ( 19 ).
- **Barts "Flank-Free" Modified Supine Position:** In this position, the patient is placed supine with modifications to allow flank-free access to the kidney, which can simplify the procedure ( 5 , 8 ).
- **Intermediate or Fully Supine Positions:** Some variations include intermediate or fully supine positions, which may offer advantages in specific patient populations or procedural preferences ( 34 ).

Abouelgreed et al. conducted a RCT comparing the GMSV and prone positions and found no significant differences in success rates, complication rates, operative time, blood loss, or the need for additional procedures ( 26 ). There is a hypothesis that higher intrarenal pressure in prone positions during PCNL may lead to increased rates of postoperative infectious complications ( 35 ). However, in ECIRS, the dual drainage through both the ureteral access sheath and the percutaneous sheath likely mitigates this risk. This dual drainage system helps maintain adequate irrigation and drainage, potentially reducing the risk of complications associated with increased intrarenal pressure. Overall, the choice of patient positioning in ECIRS should consider the specific advantages and potential risks associated with each position, aiming to optimize procedural outcomes while ensuring patient safety and comfort.

One of the primary objectives of ECIRS is to reduce the number of access tracts required during the procedure, which helps minimize intraoperative bleeding and associated risks. An additional intervention that may be considered to further mitigate the risk of bleeding is the perioperative use of tranexamic acid. It is a synthetic derivative of the amino acid lysine, known for its antifibrinolytic properties. It works by inhibiting the breakdown of fibrin clots, thereby reducing bleeding. While specific studies on the use of tranexamic acid in ECIRS are limited, its effectiveness in reducing bleeding complications has been well-documented in other surgical settings, including PCNL. In PCNL, tranexamic acid has been recommended in guidelines based on evidence from several studies and meta-analyses ( 3 , 36 ). These studies have demonstrated that tranexamic acid can effectively reduce blood loss during and after PCNL, potentially decreasing the need for blood transfusions and improving patient outcomes.

Given the similarities in procedural techniques and potential for bleeding between PCNL and ECIRS, the perioperative use of tranexamic acid in ECIRS may offer similar benefits. However, further research specifically focusing on ECIRS is necessary to establish its efficacy and safety profile in this context.

## KIDNEY ACCESS

The flexible ureteroscope used during ECIRS plays a crucial role in enhancing precision and safety by providing direct visualization and monitoring during kidney access procedures. Here are some key points regarding its benefits and recent advancements:

1. **Precise Kidney Access:** The flexible ureteroscope allows for precise localization and monitoring of the puncture site and tract dilation. By placing the ureteroscope tip in the targeted calyx, it helps guide the needle during both fluoroscopy-guided and ultrasound-guided procedures, thereby reducing puncture time and improving accuracy ( 8 , 9 ).
2. **Clinical Outcomes:** A multi-institutional retrospective cohort study by Taguchi et al demonstrated that ureteroscopy-assisted puncture reduces the risk of additional surgical interventions and decreases overall procedure time, fluoroscopy exposure, and the duration of postoperative ureteral stent placement ( 37 ).
3. **Advancements in Guidance Techniques:**
**3a - Real-time Virtual Sonography:** This technique synchronizes real-time ultrasound images with preoperative CT scans, allowing for precise localization and guidance during renal access procedures ( 38 ).
**3b - Three-Dimensional Mixed-Reality Hologram Guidance** : Emerging technologies like mixed-reality hologram guidance provide three-dimensional visualization and guidance, enhancing procedural accuracy ( 7 ).
**3c - Automated Needle Targeting with X-ray (ANT-X):** This innovative method aims to automate needle targeting using X-ray guidance, potentially improving procedural efficiency and accuracy ( 39 ). However, further research is needed to validate its effectiveness in clinical practice.

These advancements underscore the continuous evolution of ECIRS techniques towards improving outcomes and patient safety through enhanced precision, reduced procedural complexity, and optimized resource utilization. Continued research and clinical validation of these innovative approaches will be critical in further establishing their role in enhancing the efficacy and safety of ECIRS procedures.

Although not universally required, most studies in the literature describe the use of ureteral access sheaths (UAS) during flexible ureteroscopy, particularly in procedures like ECIRS. The UAS offers several advantages:

1. **Improved Kidney Drainage and Lower Intrarenal Pressure:** The presence of a UAS facilitates better drainage of the kidney during the procedure. It helps maintain a lower intrarenal pressure, which is beneficial in reducing the risk of complications such as fluid extravasation and postoperative infections ( 40 , 41 ).
2. **Facilitation of Ureteroscope Navigation:** The UAS provides a smooth pathway for the ureteroscope to navigate into the kidney. This is particularly advantageous in cases involving large-volume or impacted pelvic stones, where simultaneous lithotripsy through both antegrade and retrograde accesses can be performed effectively.
3. **Simultaneous Treatment of Stones:** In scenarios where both antegrade and retrograde accesses are utilized (as in ECIRS), the UAS allows for efficient simultaneous treatment of stones located in different parts of the kidney. This approach enhances procedural efficiency and may reduce the total operative time.

Overall, while the use of ureteral access sheaths is not mandatory, their adoption during flexible ureteroscopy, including in ECIRS, is widely recommended due to the aforementioned benefits. They contribute to improved drainage, lower intrarenal pressure, facilitate ureteroscope navigation, and enable simultaneous management of complex stone burdens, thereby enhancing the overall effectiveness and safety of the procedure.

### Tract size and equipment choice

ECIRS, similarly to PCNL, can be performed using various sizes of nephrostomy tracts. The choice of tract size is an important consideration as it can influence intraoperative bleeding and the feasibility of different lithotripsy modalities. Reducing the tract size in ECIRS may potentially minimize bleeding during the procedure. However, it's important to note that not all energy modalities used for lithotripsy are compatible with smaller endoscopes. Recent advancements in laser platforms have contributed to the trend towards instrument miniaturization, which has implications for both ECIRS and PCNL procedures. While there are no prospective studies directly comparing conventional ECIRS to mini-ECIRS, there have been two retrospective studies that have attempted to assess this comparison. Both show potential benefits such as reduced morbidity, shorter hospital stays, and faster recovery time with miniaturization. Future research, including prospective studies, is needed to systematically evaluate the advantages and limitations of mini-ECIRS compared to conventional ECIRS. This includes assessing factors such as stone clearance rate, complication rate, procedural time, and overall patient outcomes.

Usui et al. retrospectively analyzed 144 patients in matched pairs undergoing 24 or 30 Fr ECIRS versus 16.5 Fr mini-ECIRS, finding similar stone-free rate (SFR), complications and severe complications. While there was no statistically significant difference in bleeding-related complications between the groups (2.6% vs. 6.5%, p = 0.442), only the ECIRS group had cases of pseudoaneurysm or required blood transfusion. Additionally, the mini-ECIRS group experienced less pain in the perioperative period ( 42 ). Similarly, Moon et al. retrospectively compared standard (20Fr) to mini (12 Fr) ECIRS, both performed using a holmium:YAG laser for lithotripsy. Before matching, the standard ECIRS group had larger and more complex stones, as well as a higher estimated blood loss. After propensity-score matching, the only statistically significant difference that remained was the higher estimated blood loss in the standard ECIRS group ( 43 ). A meta-analysis published in 2022 by Liu et al. performed a subgroup analysis comparing mini-ECIRS to mini-PCNL. This analysis found that mini-ECIRS had a higher SFR, fewer overall and severe complications, and shorter hospital stay, while no difference was found in operative time, hemoglobin drop or blood transfusions between the two groups ( 23 ).

Vacuum-assisted procedures have recently been thoroughly studied for retrograde intrarenal surgery (RIRS) and mini-PCNL. However, only one retrospective cohort study has described the use of suctioning percutaneous sheaths in ECIRS ( 44 ). The authors reported a 91.8% final SFR after an average of 1.54 procedures for staghorn calculi. In this study authors also describe a high rate of postoperative fever, achieving 29.5%. Positive urine culture was identified as the only significant risk factor for postoperative fever, while body mass index and stone volume were significant risk factors for achieving initial stone-free status.

## SURGICAL RESULTS

Despite its more complex nature, most studies did not report longer operative time for ECIRS compared to PCNL ( 22 – 25 ). Gauhar et al. found a trend towards shorter operative time in the ECIRS group, but the difference was not statistically significant ( 24 ). Among the four meta-analyses published, only Abdullatif et al. ( 25 ) found that patients undergoing ECIRS had shorter hospital stays, while the other three reported no differences between the groups.

The evaluation of stone-free status in endourology papers indeed sparks considerable debate, primarily focusing on two key aspects: the threshold size of residual fragments and the imaging techniques employed for assessment ( 45 ). Most studies consider fragments up to 4 mm as clinically insignificant, but other cut-offs, such as 2 mm, 3 mm, or even the total absence of residual fragments are also used. The imaging techniques most employed are kidney–ureter–bladder (KUB) X-ray and/or ultrasound (US), with fewer studies using computed tomography scan (CT). The variability in follow-up durations across studies also complicates the ability to draw broad conclusions. Some studies differentiate initial and final SFR. Initial SFR refers to the evaluation after a single session of the procedure, while final SFR includes the assessment after any additional auxiliary procedures (i.e., shock wave lithotripsy, PCNL or RIRS). Recent studies have even advocated for the use of intraoperative CT during endourological procedures, though its application in ECIRS has yet to be assessed ( 46 ). Despite this variability, most papers report better initial ( 22 , 23 , 25 ) and final ( 23 ) SFRs with ECIRS. Additionally, Gauhar and Widyokirono reported lower retreatment rate in the ECIRS group in their analysis ( 22 , 24 ).

### Postoperative drainage

In a recent review encompassing 33 studies, Nedbal et al. highlighted the lack of standardization regarding the placement of postoperative nephrostomy tubes ( 47 ). Common reasons for placing nephrostomy tubes included managing bleeding, cases involving a solitary kidney, residual stones, multiple access points ( 48 ), or infection stones obstructing the calyces. However, using a nephrostomy tube may result in increased postoperative pain and delayed hospital discharge ( 49 ). Therefore, its routine use is typically not recommended unless there is a specific clinical indication. Conversely, many authors advocate for the postoperative placement of ureteral stents, especially when using a UAS.

### Complications

All four meta-analyses reported fewer complications with ECIRS compared to PCNL ( 22 – 25 ). Liu et al. categorized complications by severity and found more overall and severe complications in the PCNL group ( 23 ). The most undesired complications in endourologic percutaneous procedures are bleeding requiring transfusion, infectious events and adjacent organ injury ( 17 ). The latter is fortunately rare due to improved access techniques, as previously discussed. Gauhar et al. found a lower hemoglobin drop in the ECIRS group but similar blood transfusion rates ( 24 ), whereas Liu et al. found similar hemoglobin drop rates but lower transfusion rates ( 23 ). However, Liu et al. acknowledged that the sample size was insufficient to ensure significance and concluded that further studies are needed for a more definitive conclusion. The other two meta-analyses found no statistical difference between the groups regarding estimated blood loss and transfusion rates. Widyokirono et al. reported a significantly lower incidence of urosepsis with ECIRS compared to conventional PCNL, but no difference when compared to mini-PCNL ( 22 ). Gauhar et al. noted a trend towards a lower incidence of fever in the ECIRS group, but this was not statistically significant, and there was no difference in sepsis ( 24 ). Liu et al. also reported no difference in postoperative fever between the groups ( 23 ).

## CONCLUSIONS

In recent years, ECIRS has demonstrated significant advantages in treating large and complex kidney stones, including improved stone-free rate, reduced need for auxiliary procedures, and lower complication rate compared to traditional PCNL. Future research should focus on well-designed RCTs to provide robust evidence on the efficacy, safety, and cost-effectiveness of ECIRS, potentially establishing it as the new standard treatment.

## References

[1] Fernström I, Johansson B (1976). Percutaneous pyelolithotomy. A new extraction technique. Scand J Urol Nephrol.

[2] Assimos D, Krambeck A, Miller NL, Monga M, Murad MH, Nelson CP (2016). Surgical Management of Stones: American Urological Association/Endourological Society Guideline, PART II. J Urol.

[3] Skolarikos A, Jung H, Neisius A, Petřík A, Somani B, Tailly T (2024). EAU Guidelines on Urolithiasis.

[4] Ibarluzea G, Scoffone CM, Cracco CM, Poggio M, Porpiglia F, Terrone C (2007). Supine Valdivia and modified lithotomy position for simultaneous anterograde and retrograde endourological access. BJU Int.

[5] Bach C, Goyal A, Kumar P, Kachrilas S, Papatsoris AG, Buchholz N (2012). The Barts ‘flank-free’ modified supine position for percutaneous nephrolithotomy. Urol Int.

[6] Batagello CA, Barone Dos Santos HD, Nguyen AH, Alshara L, Li J, Marchini GS (2019). Endoscopic guided PCNL in the prone split-leg position versus supine PCNL: a comparative analysis stratified by Guy's stone score. Can J Urol.

[7] Porpiglia F, Checcucci E, Amparore D, Peretti D, Piramide F, De Cillis S (2022). Percutaneous Kidney Puncture with Three-dimensional Mixed-reality Hologram Guidance: From Preoperative Planning to Intraoperative Navigation. Eur Urol.

[8] Vicentini FC, El Hayek KKR, Szwarc M, Perrella R, Kuriki P, Cohen D (2022). Ultrasound guided endoscopic combined Intrarenal surgery - 10 steps for the success. Int Braz J Urol.

[9] Marchini GS, Lima FS, Campos MEC, Maroccolo MVO, Reggio E, Mazzucchi E (2023). Modified biplanar (0-90°) endoscopic-guided puncture technique for percutaneous nephrolithtomy: refinement with endoscopic combined intrarrenal surgery to reduce fluoroscopy and operative time. Int Braz J Urol.

[10] Bergmann J, Rosenbaum CM, Netsch C, Gross AJ, Becker B (2023). First Clinical Experience of a Novel Pulsed Solid-State Thulium:YAG Laser during Percutaneous Nephrolithotomy. J Clin Med.

[11] Patil A, Sharma R, Shah D, Gupta A, Singh A, Ganpule A (2022). A prospective comparative study of mini-PCNL using Trilogy™ or thulium fibre laser with suction. World J Urol.

[12] De Stefano V, Castellani D, Somani BK, Giulioni C, Cormio A, Galosi AB (2024). Suction in Percutaneous Nephrolithotripsy: Evolution, Development, and Outcomes from Experimental and Clinical studies. Results from a Systematic Review. Eur Urol Focus.

[13] Gücük A, Kemahlı E, Üyetürk U, Tuygun C, Yıldız M, Metin A (2013). Routine flexible nephroscopy for percutaneous nephrolithotomy for renal stones with low density: a prospective, randomized study. J Urol.

[14] Gökce Mİ, Gülpinar O, Ibiş A, Karaburun M, Kubilay E, Süer E (2019). Retrograde vs. antegrade flexible nephroscopy for detection of residual fragments following PNL: A prospective study with computerized tomography control. Int Braz J Urol.

[15] Cracco CM, Knoll T, Liatsikos EN, Osther PJ, Smith AD, Scarpa RM (2017). Rigid-only versus combined rigid and flexible percutaneous nephrolithotomy: a systematic review. Minerva Urol Nefrol.

[16] Qin P, Zhang D, Huang T, Fang L, Cheng Y (2022). Comparison of mini percutaneous nephrolithotomy and standard percutaneous nephrolithotomy for renal stones >2cm: a systematic review and meta-analysis. Int Braz J Urol.

[17] Cracco CM, Scoffone CM (2011). ECIRS (Endoscopic Combined Intrarenal Surgery) in the Galdakao-modified supine Valdivia position: a new life for percutaneous surgery?. World J Urol.

[18] Wen J, Xu G, Du C, Wang B (2016). Minimally invasive percutaneous nephrolithotomy versus endoscopic combined intrarenal surgery with flexible ureteroscope for partial staghorn calculi: A randomised controlled trial. Int J Surg.

[19] Hamamoto S, Yasui T, Okada A, Taguchi K, Kawai N, Ando R (2014). Endoscopic combined intrarenal surgery for large calculi: simultaneous use of flexible ureteroscopy and mini-percutaneous nephrolithotomy overcomes the disadvantageous of percutaneous nephrolithotomy monotherapy. J Endourol.

[20] Grasso M, Lang G, Taylor FC (1995). Flexible ureteroscopically assisted percutaneous renal access. Tech Urol.

[21] Scoffone CM, Cracco CM, Cossu M, Grande S, Poggio M, Scarpa RM (2008). Endoscopic combined intrarenal surgery in Galdakao-modified supine Valdivia position: a new standard for percutaneous nephrolithotomy?. Eur Urol.

[22] Widyokirono DR, Kloping YP, Hidayatullah F, Rahman ZA, Ng AC, Hakim L (2022). Endoscopic Combined Intrarenal Surgery vs Percutaneous Nephrolithotomy for Large and Complex Renal Stone: A Systematic Review and Meta-Analysis. J Endourol.

[23] Liu YH, Jhou HJ, Chou MH, Wu ST, Cha TL, Yu DS (2022). Endoscopic Combined Intrarenal Surgery Versus Percutaneous Nephrolithotomy for Complex Renal Stones: A Systematic Review and Meta-Analysis. J Pers Med.

[24] Gauhar V, Castellani D, Cracco CM, Scoffone CM, Lim EJ, Rubilotta E (2022). Is endoscopic combined intrarenal surgery ready for primetime in endourology? Outcomes from a systematic review and meta-analysis. Cent European J Urol.

[25] Abdullatif VA, Sur RL, Abdullatif ZA, Szabo SR, Abbott JE (2022). The Safety and Efficacy of Endoscopic Combined Intrarenal Surgery (ECIRS) versus Percutaneous Nephrolithotomy (PCNL): A Systematic Review and Meta-Analysis. Adv Urol.

[26] Abouelgreed TA, Abdelaal MA, Amin MM, Elatreisy A, Shalkamy O, Abdrabuh AM (2022). Endoscopic combined intrarenal surgery in the prone split-leg position versus Galdakao-modified supine Valdivia position for the management of partial staghorn calculi. BMC Urol.

[27] Estrade V, Meria P, Almeras C, Lithiasis Committee of the French Association of Urology (CLAFU) (2023). 2022 Recommendations of the AFU Lithiasis Committee: Combined approach for the management of kidney and ureteral stones (Endoscopic Combined IntraRenal Surgery, ECIRS). Prog Urol.

[28] Tanidir Y, Sekerci CA, Genc YE, Gokmen E, Arslan F, Yucel S (2024). Endoscopic combined intrarenal surgery versus percutaneuos nephrolithotomy for complex pediatric stone disease: A comparative analysis of efficacy and safety. J Pediatr Urol.

[29] Sugino F, Nakane K, Kawase M, Iinuma K, Kawase K, Koie T (2023). Endoscopic combined intrarenal surgery for renal allograft lithiasis using "sheath-connection technique": A case report. Urol Case Rep.

[30] Tsaturyan A, Faria-Costa G, Peteinaris A, Lattarulo M, Martinez BB, Vrettos T (2023). Endoscopic management of encrusted ureteral stents: outcomes and tips and tricks. World J Urol.

[31] Limudomporn P, Phengsalae Y, Ketsuwan C (2022). A giant ureteric calculus successfully removed by mini-endoscopic combined intrarenal surgery: A case report. Urol Case Rep.

[32] Manikandan R, Mittal JK, Dorairajan LN, Mishra AK, Sreerag KS, Verma A (2016). Endoscopic Combined Intrarenal Surgery for Simultaneous Renal and Ureteral Stones: A Retrospective Study. J Endourol.

[33] Grande MS, Campobasso D, Inzillo R, Moretti M, Facchini F (2021). The use of endoscopic combined intrarenal surgery as an additional approach to upper urinary tract urothelial carcinoma: Our Experience. Indian J Urol.

[34] Jung HD, Kim JC, Ahn HK, Kwon JH, Han K, Han WK (2018). Real-time simultaneous endoscopic combined intrarenal surgery with intermediate-supine position: Washout mechanism and transport technique. Investig Clin Urol.

[35] Perrella R, Vicentini FC, Paro ED, Torricelli FCM, Marchini GS, Danilovic A (2022). Supine versus Prone Percutaneous Nephrolithotomy for Complex Stones: A Multicenter Randomized Controlled Trial. J Urol.

[36] Cleveland B, Norling B, Wang H, Gandhi V, Price CL, Borofsky MS (2023). Tranexamic acid for percutaneous nephrolithotomy. Cochrane Database Syst Rev.

[37] Taguchi K, Yamashita S, Hamamoto S, Deguchi R, Kawase K, Okada T (2021). Ureteroscopy-assisted puncture for ultrasonography-guided renal access significantly improves overall treatment outcomes in endoscopic combined intrarenal surgery. Int J Urol.

[38] Hamamoto S, Unno R, Taguchi K, Ando R, Hamakawa T, Naiki T (2017). A New Navigation System of Renal Puncture for Endoscopic Combined Intrarenal Surgery: Real-time Virtual Sonography-guided Renal Access. Urology.

[39] Taguchi K, Hamamoto S, Kato T, Iwatsuki S, Etani T, Okada A (2021). Robot-assisted fluoroscopy-guided renal puncture for endoscopic combined intrarenal surgery: a pilot single-centre clinical trial. BJU Int.

[40] Doizi S, Uzan A, Keller EX, De Coninck V, Kamkoum H, Barghouthy Y (2021). Comparison of intrapelvic pressures during flexible ureteroscopy, mini-percutaneous nephrolithotomy, standard percutaneous nephrolithotomy, and endoscopic combined intrarenal surgery in a kidney model. World J Urol.

[41] Santa Cruz JAC, Danilovic A, Vicentini FC, Brito AH, Batagello CA, Marchini GS (2024). Ureteral access sheath. Does it improve the results of flexible ureteroscopy? A narrative review. Int Braz J Urol.

[42] Usui K, Komeya M, Taguri M, Kataoka K, Asai T, Ogawa T (2020). Minimally invasive versus standard endoscopic combined intrarenal surgery for renal stones: a retrospective pilot study analysis. Int Urol Nephrol.

[43] Moon YJ, Cho KS, Jung DC, Chung DY, Lee JY (2023). The Consecutive 200 Cases of Endoscopic-Combined Intrarenal Surgery: Comparison between Standard and Miniature Surgeries. Medicina (Kaunas).

[44] Tominaga K, Inoue T, Yamamichi F, Fujita M, Fujisawa M (2023). Impact of Vacuum-Assisted Mini-Endoscopic Combined Intrarenal Surgery for Staghorn Stones: Analysis of Perioperative Factors of Postoperative Fever and Stone-Free Status. J Endourol.

[45] Higgins AM, Ganesan V, Ghani KR, Agarwal DK, Borofsky MS, Dauw CA (2022). The 2023 Stone-Free CT Mandate: Addressing the Two Sides of the Debate. J Endourol.

[46] Lepine HL, Vicentini FC, Mazzucchi E, Molina WR, Marchini GS, Torricelli FC (2024). Intraoperative computed tomography for detection of residual stones in endourology procedures: systematic review and meta-analysis. Int Braz J Urol.

[47] Nedbal C, Jahrreiss V, Cerrato C, Castellani D, Kamal WK, Hameed Z (2023). Current role of endoscopic combined intrarenal surgery in the management of renal stones: A scoping review. Indian J Urol.

[48] Batagello CA, Vicentini FC, Monga M, Miller AW, Marchini GS, Torricelli FCM (2022). Tranexamic acid in patients with complex stones undergoing percutaneous nephrolithotomy: a randomised, double-blinded, placebo-controlled trial. BJU Int.

[49] Isac W, Rizkala E, Liu X, Noble M, Monga M (2014). Tubeless percutaneous nephrolithotomy: outcomes with expanded indications. Int Braz J Urol.

[50] Nuño de la Rosa I, Palmero JL, Miralles J, Pastor JC, Benedicto A (2014). A comparative study of percutaneous nephrolithotomy in supine position and endoscopic combined intrarenal surgery with flexible instrument. Actas Urol Esp.

[51] Leng S, Xie D, Zhong Y, Huang M (2018). Combined Single-Tract of Minimally Percutaneous Nephrolithotomy and Flexible Ureteroscopy for Staghorn Calculi in Oblique Supine Lithotomy Position. Surg Innov.

[52] Zhao F, Li J, Tang L, Li C (2021). A comparative study of endoscopic combined intrarenal surgery (ECIRS) in the galdakao-modified supine valdivia (GMSV) position and minimally invasive percutaneous nephrolithotomy for complex nephrolithiasis: a retrospective single-center study. Urolithiasis.

[53] Kontos S, Papatsoris A, Nalagatla SK (2018). ECIRS (endoscopic combined intrarenal surgery) versus fluoroscopic-guided renal access during supine percutaneous nephrolithotomy (PCNL): A comparative study. [Internet]. Hell Urol.

[54] Isac W, Rizkala E, Liu X, Noble M, Monga M (2013). Endoscopic-guided versus fluoroscopic-guided renal access for percutaneous nephrolithotomy: a comparative analysis. Urology.

[55] Xu K, Li Z Comparison of Multi-tract minimally invasive percutaneous nephrolithotomy and Endoscopic Combined Intrarenal Surgery for Staghorn Renal Calculi: A single institution experience.

[56] Mami D, Alchinbayev M, Kazachenko A (2021). Comparison of minimally invasive treatment methods for urinary stones: A retrospective analysis. [Internet]. Electron J Gen Med.

[57] Kawahara T, Ito H, Terao H, Yoshida M, Ogawa T, Uemura H (2012). Ureteroscopy assisted retrograde nephrostomy: a new technique for percutaneous nephrolithotomy (PCNL). BJU Int.

[58] Gauhar V, Castellani D, Cracco CM, Scoffone CM, Lim EJ, Rubilotta E (2022). Is endoscopic combined intrarenal surgery ready for primetime in endourology? Outcomes from a systematic review and meta-analysis. Cent European J Urol.

[59] Gao H, Zhang H, Wang Y, Li K, Du W, Wang X (2019). Treatment of Complex Renal Calculi by Digital Flexible Ureterorenoscopy Combined with Single-Tract Super-Mini Percutaneous Nephrolithotomy in Prone Position: A Retrospective Cohort Study. Med Sci Monit.

[60] Beck S, Jain N, Kaplan A, Box G, Clayman R, Mcdougall E (2009). Endoscopic guided percutaneous access during percutaneous nephrolithotomy: Is it of any clinical advantage?. J Endourol.

[61] Zelvys A, Cekauskas A, Jankevicius F (2014). B71 - Role of combined intrarenal surgery (ECIRS) in management of large/complex kidney stones. Eur Urol Suppl.

[62] Zhang W, Lu P (2016). Super-mini percutaneous nephrolithotomy combined with flexible ureteroscopy versus the Chinese minimally invasive percutaneous nephrolithotomy: An efficacy analysis in patients with renal stones 2.5-4.0 cm. J Endourol.

[63] Yong D, Koh S, Tan Y (2017). Supine percutaneous nephrolithotomy is safe and achieves higher stone free rates when combined with retrograde intrarenal surgery (endoscopic combined intra renal surgery). J Endourol.

[64] Kavaliauskaite R, Želvys A (2018). 65 – Endoscopic combined intrarenal surgery value in multiple renal stones treatment. Eur Urol Suppl.

